# The Role of Artificial Intelligence in Combatting Respiratory Tract Infections

**DOI:** 10.7759/cureus.63635

**Published:** 2024-07-01

**Authors:** Vasiliki E Georgakopoulou

**Affiliations:** 1 Department of Pathophysiology/Pulmonology, Laiko General Hospital, Athens, GRC

**Keywords:** public health, personalized medicine, diagnostics, respiratory tract infections, artificial intelligence

## Abstract

Respiratory tract infections (RTIs) such as pneumonia, bronchitis, and COVID-19 are significant global health concerns due to their high morbidity and mortality rates. The advent of artificial intelligence (AI) offers innovative solutions across various aspects of RTI management, including diagnosis, prediction, treatment, and prevention. AI algorithms enhance diagnostic accuracy by analyzing extensive data from electronic health records and imaging studies, often surpassing human radiologists in identifying diseases such as pneumonia. For instance, AI-based image recognition tools have demonstrated remarkable precision in detecting pneumonia from chest X-rays. Additionally, AI models can predict disease outbreaks and optimize public health responses, as exemplified during the COVID-19 pandemic where AI predicted infection hotspots and evaluated the effectiveness of containment measures. In personalized medicine, AI tailors treatments based on individual patient profiles, thereby improving therapeutic outcomes and accelerating drug discovery. Wearable AI devices facilitate early detection and prevention of RTIs through continuous health monitoring. Despite its transformative potential, AI implementation in healthcare faces challenges, including data privacy, algorithm transparency, and ethical concerns. Addressing these issues necessitates collaboration among technologists, healthcare providers, and policymakers to ensure responsible and equitable integration of AI technologies. This editorial underscores the transformative potential of AI in managing RTIs and calls for robust frameworks to harness AI's benefits while safeguarding patient rights.

## Editorial

Respiratory tract infections (RTIs), including illnesses such as pneumonia, bronchitis, and COVID-19, pose significant global health challenges. The high morbidity and mortality rates associated with these infections demand innovative solutions to improve diagnosis, treatment, and prevention. In recent years, artificial intelligence (AI) has emerged as a powerful tool in the fight against RTIs, offering unprecedented capabilities in data analysis, predictive modeling, and personalized medicine.

One of the most promising applications of AI in managing RTIs is in the realm of diagnostics. Traditional diagnostic methods often rely on clinical judgment and standard laboratory tests, which can be time-consuming and sometimes inconclusive. AI algorithms, however, can analyze vast amounts of data from various sources, including electronic health records, imaging studies, and even genomic data, to identify patterns and make accurate predictions. For instance, AI-based image recognition tools have shown remarkable accuracy in detecting pneumonia from chest X-rays, often outperforming human radiologists. A systematic review and meta-analysis demonstrated that AI models for chest imaging could diagnose COVID-19 and other pneumonias with high precision, significantly aiding clinical decision-making [1]. The study systematically reviewed and meta-analyzed the diagnostic accuracy and methodological quality of AI models for distinguishing COVID-19 from other pneumonias using chest imaging. By searching multiple databases and applying rigorous quality assessment tools, including QUADAS-2, Radiomics Quality Score (RQS), and the Checklist for Artificial Intelligence in Medical Imaging (CLAIM) checklist, the study included 32 relevant studies encompassing 6737 participants. The meta-analysis demonstrated that AI models achieved a pooled area under the curve (AUC) of 0.96, with a sensitivity of 0.92 and specificity of 0.91, indicating high diagnostic accuracy [1].

In addition to diagnostics, AI plays a crucial role in predicting disease outbreaks and managing public health responses. Machine learning models can process epidemiological data to forecast the spread of respiratory infections, enabling health authorities to implement targeted interventions. During the COVID-19 pandemic, AI was instrumental in predicting infection hotspots and evaluating the effectiveness of containment measures. For example, a deep learning-based triage and analysis system for COVID-19 demonstrated high accuracy in diagnosing the disease across different populations, thus optimizing resource allocation and planning vaccination campaigns [2].

Treatment of RTIs also benefits significantly from AI innovations. Personalized medicine, which tailors treatment plans based on individual patient characteristics, is becoming increasingly feasible with AI. Machine learning algorithms can analyze a patient's genetic makeup, lifestyle factors, and response to previous treatments to recommend the most effective therapeutic strategies. For instance, AI-driven platforms can suggest the best antibiotic regimen for bacterial infections, minimizing the risk of antibiotic resistance and improving patient outcomes. AI can also assist in drug discovery by identifying potential antiviral compounds and predicting their efficacy, thus accelerating the development of new treatments [3].

AI's role extends beyond acute management to the prevention of RTIs. Wearable devices equipped with AI can monitor patients' vital signs and detect early symptoms of respiratory infections, prompting early intervention and reducing the likelihood of severe illness. These devices can also provide valuable data for continuous health monitoring, helping to identify at-risk populations and implement preventive measures. AI-driven health education programs can further enhance public awareness and encourage behaviors that reduce the spread of infections, such as proper hand hygiene and vaccination adherence [4].

Despite the immense potential of AI, there are challenges and ethical considerations that need to be addressed. Ensuring the privacy and security of health data is paramount, as is maintaining transparency in AI algorithms to avoid biases that could affect clinical decisions. Collaboration between technologists, healthcare providers, and policymakers is essential to create robust frameworks that harness AI's benefits while safeguarding patient rights. Issues such as the need for high-quality training data, external validation of models, and the management of AI system errors and biases must be carefully navigated [5].

In conclusion, AI holds transformative potential in addressing the complexities of RTIs. From enhancing diagnostic accuracy and predicting outbreaks to personalizing treatments and preventing disease, AI offers innovative solutions that can significantly improve public health outcomes. As we continue to integrate AI into healthcare, it is crucial to navigate the associated challenges with a focus on ethical and equitable implementation. By doing so, we can harness the full power of AI to combat RTIs and ultimately save lives.
